# Image texture and radiation dose properties in CT

**DOI:** 10.1120/jacmp.v17i3.5900

**Published:** 2016-05-08

**Authors:** Dawid Mozejko, Hilde Kjernlie Andersen, Marius Pedersen, Dag Waaler, Anne Catrine Trægde Martinsen

**Affiliations:** ^1^ Faculty of Computer Science and Media Technology, Gjøvik University College Gjøvik Norway; ^2^ The Intervention Centre, Oslo University Hospital Oslo Norway; ^3^ Faculty of Health, Care and Nursing Gjøvik University College Gjøvik Norway; ^4^ The Department of Physics University in Oslo Norway

**Keywords:** CT, image texture, texture, radiation dose, noise power spectrum, noise

## Abstract

The aim of this study was to compare image noise properties of GE Discovery HD 750 and Toshiba Aquilion ONE. The uniformity section of a Catphan 600 image quality assurance phantom was scanned with both scanners, at different dose levels and with extension rings simulating patients of different sizes. 36 datasets were obtained and analyzed in terms of noise power spectrum. All the results prove that introduction of extension rings significantly altered the image quality with respect to noise properties. Without extension rings, the Toshiba scanner had lower total visible noise than GE (with GE as reference: FC18 had 82% and FC08 had 80% for 10 mGy, FC18 had 77% and FC08 74% for 15 mGy, FC18 had 80% and FC08 77% for 20 mGy). The total visible noise (TVN) for 20 and 15 mGy were similar for the phantom with the smallest additional extension ring, while Toshiba had higher TVN than GE for the 10 mGy dose level (120% FC18, 110% FC08). For the second and third ring, the GE images had lower TVN than Toshiba images for all dose levels (Toshiba TVN is greater than 155% for all cases). The results indicate that GE potentially has less image noise than Toshiba for larger patients. The Toshiba FC18 kernel had higher TVN than the Toshiba FC08 kernel with additional beam hardening correction for all dose levels and phantom sizes (120%, 107%, and 106% for FC18 compared to 110%, 98%, and 97%, for FC08, for 10, 15 and 20 mGy doses, respectively).

PACS number(s): 87.57.Q‐, 87.57.nf, 87.57.C‐

## I. INTRODUCTION

Computed tomography (CT) is widely used for medical diagnostic purposes. The CT usage worldwide has increased rapidly in the last decades.^1^, ^2^ CT is the imaging modality with the highest radiation dose among medical radiography techniques,^(3)^ and CT examinations may increase the additional risk of cancer for patients.^4^, ^5^, ^6^ Thus, the radiation dose should be as low as possible while at the same time, obtaining the diagnostic information vital for patient safety.^(7)^ Therefore, all the CT vendors have improved the CT technology to improve image quality and reduce radiation dose over the last decade. However, the different vendors have different scanner designs, different technological platforms, and different reconstruction algorithms, resulting in different image quality at equal dose level, even if the scan parameter settings are as similar as possible and the scanned object is the same.

It is important to realize that details of the image reconstruction algorithms and reconstruction kernels are kept private by the vendors. The radiographers and radiologists receive reconstruction filter recommendations from the vendors, based on different indications and anatomical areas of interest. As new CT scanners are introduced in a radiological department in a hospital, optimizing the image quality can be challenging because the image texture is different for different scanners. Often, the image texture of the old scanner is familiar to the radiologists, and the new scanner represents a new image texture to which they must get accustomed. In addition, new reconstruction methods are introduced without all necessary information about the functionality, so it might be difficult to choose which type of reconstruction kernel is the most advantageous for a given specific examination. Image noise is one of the critical factors with respect to soft‐tissue lesion detectability. The aim of this study was to compare image noise texture for different reconstruction kernels, different dose levels, and different phantom diameters for two different CT scanners to objectively evaluate differences in image noise properties between these two CT scanners.

## II. MATERIALS AND METHODS

Two CT scanners were used in the study: GE Discovery HD 750 (GE Healthcare, Milwaukee, WI) and Toshiba Aquilion ONE (Toshiba Medical Systems, Tokyo, Japan). For short, they will be referred to as GE and Toshiba, respectively.

**Figure 1:**
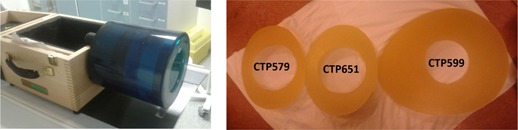
On the left Catphan 600 Phantom and on the right Catphan 600 CTP anthropomorphic annuli.

The “Image uniformity module” (CTP 486) in a commercially available image quality phantom, Catphan 600 (The Phantom Laboratory, Salem, NY),^(8)^ was used to measure image noise levels and image texture. Additional annuli (later referred to as rings) were mounted outside the phantom, to simulate patients of different sizes (Fig. 1). CT scans were performed at three different dose levels (${\text{CTDI}}_{\text{vol}}$ 10, 15, and 20 mGy) on both scanners. On all dose levels, scans were performed for Catphan 600 and for Catphan 600 with additional rings. These rings were of oval shape, with following dimensions: CTP579 — 25–35 cm oval OD uniformity material body annulus; CTP651 — 30–38 cm oval OD uniformity material body annulus; CTP599 — 45–55 cm oval OD uniformity material body annulus. These are referred to as the first ring, second ring, and third ring.

All parameter settings are listed in Table 1. The scan parameters used, were as similar as possible for the two scanners, in order to compare the reconstruction kernels and noise properties.

**Table 1:** Scan settings used in experiment. All scans were done in axial mode.

*Scanner*	*GE*	*Toshiba*
kVp	120	120
CTDI (mGy)	10, 15, 20	10, 15, 20
reconstructed FOV (mm)	210, 360, 400, 500	210, 360, 400, 500
slice thickness (mm)	5	5
number of slices	4	4
image matrix size (pix)	512×512	512×512
convolution kernels	STANDARD	FC08, FC18

A noise power spectrum (NPS) presents the noise distribution over all spatial frequencies, and will indicate whether the noise texture is coarser or grainier structured. The formula used for NPS was:

(1) $$NPS(u_{n},v_{k})={\text{lim}}_{N_{x,}N_{y}\to \infty }(N_{x}N_{y}\Delta x\Delta y)〈{\left|FT_{nk}[I(x,y)-\bar{I}]\right|}^{2}〉,$$

where *FT* represents two dimensional Fourier Transform, *u* and *v* are spatial frequency $\left[{\text{mm}}^{-1}\right]$ in X‐Y directions, $\bar{I}$ is the mean pixel value across the ROI (subtracted from each pixel to remove dc component and reduce artifacts), and *I*(*x, y*) includes the CT number at pixel location (x,y).^(9)^

**Figure 2:**
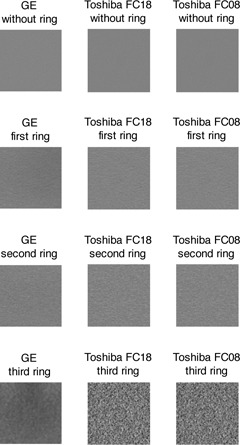
Comparison of ROI for the different scanners and with different rings for 20 mGy dose level. All ROIs are shown with the same range.

**Figure 3:**
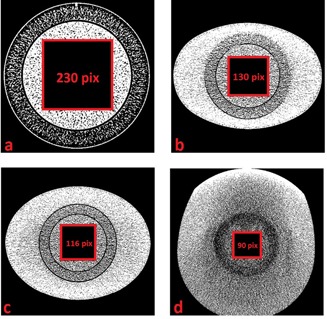
The dimensions of the ROIs matrix sizes were adjusted to cover the same object size in millimeters independent of the addition of rings.

The ROI was placed in the uniform, central part of the phantom. Figure 2 shows a comparison of ROI for the different scanners and with different rings for 20 mGy dose level. All images had a matrix of 512×512 pixels. As the displayed field of view (DFOV) increased with the addition of rings, the single pixel size was increasing. Thus, the dimensions of the ROIs matrix sizes were adjusted in order to keep them constant in object size (millimeters) (Fig. 3, 1, 2).

**Figure 4:**
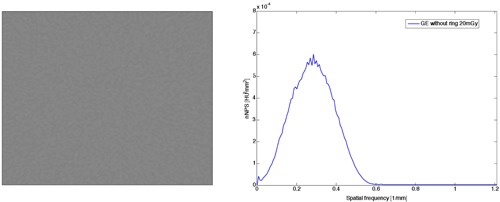
The ROI area (left) and the radially averaged one‐dimensional NPS (right) for GE without a ring for 20 mGy dose.

Two cases of NPS were analyzed. In order to achieve removal of systematic noise, the NPS was calculated as follows. The NPS of four slices were calculated separately. These four NPS were averaged, resulting in one averaged NPS dataset. The two‐dimensional NPS spectra were radially averaged to provide possibility to compare the shapes of the NPS spectra. An example is shown in Fig. 4.

**Table 2:** FOV changes with the introduction of the rings, and the effect they have on the ROI size in pixels.

	*DFOV (mm)*	*Limit (pixels)*	*ROI Size (pixels)*
Without ring	210	243.8	230
First ring	360	142.2	130
Second ring	397	129.0	115
Third ring	500	102.4	90

**Figure 5:**
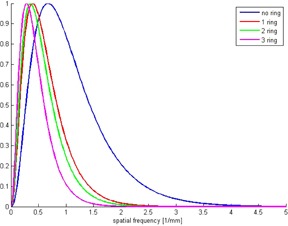
One‐dimensional human visual response curves without an additional ring and for the different rings.

Furthermore, the one‐dimensional NPS curves were filtered with a human visual response curve,^9^, ^10^ and adjusted according to display fields of view:

(2) $$NPS_{eye}=NPS(r)\cdot {\left|V(\rho )\right|}^{2},$$

(3) $$\rho =r\cdot \frac{FOV\cdot R\cdot \pi }{D\cdot 180},$$

(4) $$V(\rho )={\left|\eta \rho^{al}\cdot e^{-a^{2}\rho^{a3}}\right|}^{2},$$

where *r* is the spatial frequency in ${\text{mm}}^{-1}$, ρ is the radial spatial frequency seen by an observer (cycles per degree), *FOV is* the field of view in mm, *R* is the viewing distance, *D* is the displayed image in mm, η is a normalizing factor to ensure that V(ρ) is set to 1 at its maximum, and the parameters $a^{1}$, $a^{2}$, $a^{3}$ are set to 1.5, 0.98, and 0.68. A viewing distance R of 40 cm and display size D of 30 cm was used in this study. The resultant curves present the sensitivity of standard human observer for the noise present in the ROI (Fig. 5). Peak frequency of ${\text{NPS}}_{\text{eye}}$, which is the frequency at which its peak value was,^(9)^ is computed by fitting a third degree polynomial to ${\text{NPS}}_{\text{eye}}$, and finding the point where the derivative is equal to zero.

After obtaining the ${\text{NPS}}_{\text{eye}}$, the root mean square difference (RMSD) was calculated for the datasets, using the equation described by Armstrong and Collopy:^(11)^

(5) $$RMSD=\sqrt{\frac{1}{N}\sum_{i=1}^{N}{\left(D_{1}-D_{2}\right)}^{2},}$$

where *N* is the number of samples, $D_{1}$ is the first dataset, $D_{2}$ is the dataset that is compared to $D_{1}$. If the RMSD equals zero, the two datasets are equal. A higher RMSD indicates that there is a difference between $D_{1}$ and $D_{2}$.

Data from the Toshiba datasets with convolution kernels FC18 and FC08 (without and with compensation for beam hardening effect, respectively) were compared to the GE standard datasets. The higher the RMSD, the more different the Toshiba datasets are from GE datasets (and vice versa). All comparisons were performed at the same dose levels and with the same phantom size.

**Figure 6:**
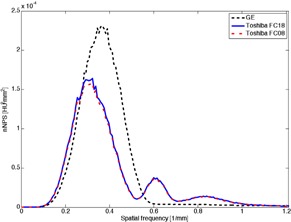
${\text{NPS}}_{\text{eye}}$ for Toshiba FC08, FC18, and GE for 20 mGy dose without rings.

**Figure 7:**
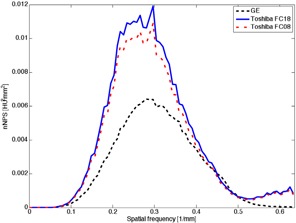
${\text{NPS}}_{\text{eye}}$ for Toshiba FC08, FC18, and GE for the second ring and 20 mGy dose.

The ${\text{NPS}}_{\text{eye}}$ presents the total visible noise spectra, and thereby gives an opportunity to compare the scanners with respect to visual noise. An example of ${\text{NPS}}_{\text{eye}}$ with a ring is shown in Fig. 6, and with the second ring in Fig. 7. In order to compare numerous curves, the area under curves was used as a measure representing the total visible noise (TVN):

(6) $$TVT=\int d\Theta \int NPS_{ID}f_{r}df_{r},$$

where $f_{r}$ is the spatial frequency.

TVN gives simple one‐value information about the amount of the noise in the dataset, and was used to organize the datasets.

## III. RESULTS

The NPS peak frequencies for different phantom sizes and 20 mGy dose level for the GE scanner and the Toshiba scanner (see Table 3) indicates that, without extension rings, the noise texture for the GE scanner is grainier compared to that of the Toshiba scanner. As the phantom diameter increased, the image texture for both scanners became coarser. The change was larger for GE than Toshiba, and the Toshiba scanner had the grainiest noise for the largest ring.

**Figure 8:**
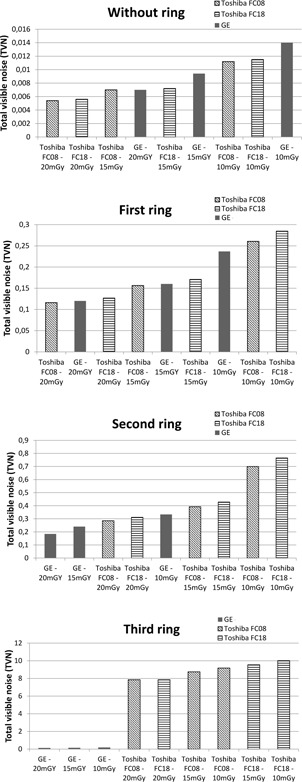
Comparison of total visible noise (TVN) for GE and Toshiba FC08 and FC18 for different doses without a ring, first ring, second ring, and third ring.

For the phantom without additional rings, the Toshiba scanner showed the smallest total amount of visible noise (TVN) compared to GE for all dose levels (with GE as a reference with 100%, FC18 had 82%, and FC08 had 80% for 10 mGy, FC18 had 77% and FC08 74% for 15 mGy, FC18 had 80% and FC08 77% for 20 mGy, as can be seen in Table 4). There was no observable difference in TVN between the reconstruction kernels with and without beam hardening correction for the Toshiba scanner without additional rings. For the phantom with the smallest additional extension ring, the GE scanner and the Toshiba scanner had similar TVN for 20 and 15 mGy, while Toshiba images had higher TVN than GE for the 10 mGy dose level (FC18 had 120% and FC08 had 110%). For this phantom diameter, the FC18 had higher TVN than the TC08 for the Toshiba scanner (120%, 107%, and 106% for FC18 compared to 110%, 98%, and 97% for FC08, for 10, 15, and 20 mGy dose, respectively). For the second and third ring, the GE images had lower TVN than the Toshiba images for all dose levels (Toshiba TVN is greater than 155% for all cases). In some cases, the TVN was even lower for lower dose GE images than for the higher dose Toshiba images, as can be seen in Fig. 8. The FC18 had generally higher TVN than FC08 also for these phantom diameters. Differences of percentages for the different scanners, doses, and rings are shown in Table 4, where GE is used as a reference.

**Figure 9:**
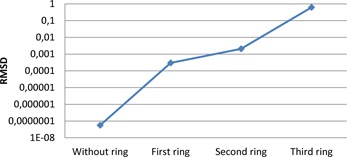
Comparison of Toshiba standard protocols FC18 and FC08 (without and with compensation for beam hardening effect, respectively) shows that the difference is larger with increasing phantom diameter.

The comparison of Toshiba standard protocols FC18 and FC08, without and with compensation for beam hardening effect, respectively, was done both without and with human visual response filtering of the NPS. In the evaluation of NPS, one can realize that the differences between the reconstruction kernels were minimal. The noise magnitude increased with lower dose, and with larger phantom size, but the NPS curves had very similar shapes. The differences were negligibly small for small rings. The TVN was larger for FC18 than for FC08 for all phantom diameters except the smallest one, as seen in Fig. 8. On the other hand, the peak frequency was exactly the same for both kernels, indicating that the image texture is the same, independent of reconstruction kernel. This result indicates that the compensation for the beam hardening effect in the FC filters from Toshiba has some influence on the amount of image noise for large objects. The results can be seen in Table 3 and Fig. 9.

**Table 3:** Comparison of peak frequency in 1/mm between GE and Toshiba with different rings at 20 mGy dose.

	*No Ring Peak Frequency*	*First Ring Peak Frequency*	*Second Ring Peak Frequency*	*Third Ring Peak Frequency*
GE	0.37	0.32	0.28	0.18
Toshiba FC18	0.32	0.27	0.26	0.25
Toshiba FC08	0.30	0.28	0.27	0.26

**Table 4:** Differences in TVN in percentages between the different scanners, dose, and rings. GE is used as a reference for comparison (100%).

	*Without Ring*			*First Ring*			*Second Ring*			*Third Ring*		
Scanner/Dose	10 mGy	15 mGy	20 mGy	10 mGy	15 mGy	20 mGy	10 mGy	15 mGy	20 mGy	10 mGy	15 mGy	20 mGy
Toshiba FC18	82%	77%	80%	120%	107%	106%	230%	179%	169%	5334%	6334%	6660%
GE	100%	100%	100%	100%	100%	100%	100%	100%	100%	100%	100%	100%
Toshiba FC08	80%	74%	77%	110%	98%	97%	210%	164%	155%	4889%	5802%	6097%

**Figure 10:**
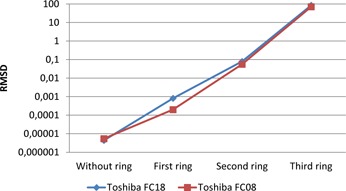
RMSD based on the ${\text{NPS}}_{\text{eye}}$ values was calculated for different GE and Toshiba kernels. GE standard kernel was compared with both Toshiba FC18 and FC08. The lower the values, the more similar were the kernels.

For increasing phantom sizes, the NPS spectra for GE and Toshiba became increasingly deviant. Interestingly, the FC08 kernel corresponded better to the GE standard kernel as the phantom diameter was increasing, indicating that the additional beam hardening for this filter corresponds to the beam hardening correction in GE's standard kernel. The results are shown in Fig. 10.

## IV. DISCUSSION

The NPS measurements show that the image noise texture varied between the two scanners tested. Toshiba had a more coarse noise pattern than GE for the small phantoms tested, which might also support the radiologist's impressions of the images as the Toshiba scanner was installed in the hospital. These findings are also supported by Singh et al.^(12)^

For the smallest phantom diameter, the Toshiba scanner had the lowest TVN for all dose levels compared to the GE scanner, but as the phantom diameter increased, the TVN increased more for the Toshiba scanner compared to the GE scanner. These results indicate that the GE scanner is compensating better for increased patient sizes, and in fact, for the phantom with the second extension ring, GE has less visible noise for 15 mGy than Toshiba has for 20 mGy. Correspondingly, 10 mGy dose level for the GE scanner resulted in lower TVN than 15 mGy for the Toshiba scanner. For the largest phantom diameter, GE outperforms Toshiba with respect to total visible noise for all dose levels.

There is a small additional peak in the NPS in the higher frequency region in the Toshiba images. In those images a weak ring artifacts were present in the center. Toshiba has ring artifact corrections in their algorithms, but still there might have been some weak artifacts in the center of the images. The second peak of this image becomes more visible after filtering with the human visual response function, as the human visual system is more sensitive in this frequency region.

The FC08 datasets have lower TVN than FC18. The results for FC08 and FC18 were favoring FC08, which was the expected result, because with increasing size, increased beam hardening would disrupt the image and FC08 should compensate for this effect better than FC18. This shows that the compensation for the beam hardening has some effect on the images of larger objects. Only the datasets without any extension ring were spatially symmetrical, due to ellipsoid shape of the rings. The datasets were compared within the same phantom size, and thereby had similar spatial distributions of NPS. Due to the decreasing number of pixels within the ROIs with increasing size of the rings, radial averaging was performed to preserve sufficient amount of data for averaging and obtaining smooth curves. For the largest phantom diameter, a different method of representing 1D NPS, like averaging from line/few lines of pixels would provide very little information. Although it is common in literature to use few ROIs from each slice,^9^, ^13^, ^14^ this method was not used in this study in order to achieve smoothness of the NPS of phantom with increasing phantom diameter. With such division, the resultant NPS has less samples, and normally, more noise. This becomes more pronounced and problematic with increasing phantom diameters, since the number of samples decreases either way.

The ${\text{NPS}}_{\text{eye}}$ presents the TVN, and thereby gives an opportunity to compare datasets with respect to visual noise. This is the information that could be directly related to the impression that the observers have about the quality of the image, and his/her ability to see the diagnostically significant features. In Fig. 5, the change of human visual response function for different phantom sizes can be seen. This occurred due to different FOV used for different rings, since FOV is one of the parameters of the equation for human visual response function.

This study had limitations. First of all, only image noise properties were evaluated, not spatial resolution or other image quality parameters. To fully evaluate the image quality for different scanners and different reconstruction techniques, both noise properties and spatial resolution should be considered. The TVN calculations were only performed for 40 cm distance. The GE and Toshiba reconstruction kernels may be affected by FOV, and this might have affected the results. Still, the only possible way to compare the image quality for different CT scanners is by using as similar a reconstruction technique as possible, and therefore the abdominal filters recommended by the vendors were used in this study. For the TVN measurements, the distance is important. Other distances would have given other results. Still, in this study relative differences between different reconstruction filters and different scanners were performed. For all measurements, the distance was the same. For NPS analysis, introduction of extension rings decreased the number of pixels in the ROIs used for NPS calculation. Decreased numbers of pixels might have influenced the NPS analysis. Still, the same method was used for all measurements and the results were compared relatively to each other.

## V. CONCLUSIONS

The results indicate that the noise texture is different for the two scanners in this study. For small objects, Toshiba had a more coarse noise pattern than GE, while GE had a coarser noise pattern than Toshiba for the largest objects. Toshiba's beam hardening correction filter improved the noise properties as the phantom size increased, compared to the filter without this correction. Overall, the GE scanner had less total visible noise compared to the Toshiba scanner, except for the smallest phantom diameter. This means that the GE scanner will produce CT images with less noise for normal to larger patient sizes compared to the Toshiba scanner, and thereby potentially give better diagnostic information.

## COPYRIGHT

This work is licensed under a Creative Commons Attribution 4.0 International License.

## Supporting information

Supplementary Material

Supplementary Material

Supplementary Material

Supplementary Material

Supplementary Material

Supplementary Material

Supplementary Material

Supplementary Material

Supplementary Material

Supplementary Material

Supplementary Material

## References

[1] Kalender WA . Computed tomography: fundamentals, system technology, image quality, applications. s.l. Munich: Publicis MCD Vertag; 2000.

[2] Smith‐Bindman R , Lipson J , Marcus EAR , et al. Radiation dose associated with common computed tomography examinations and the associated lifetime attributable risk of cancer. Arch Intern Med. 2009;169(22):2078–86. 20008690 10.1001/archinternmed.2009.427 PMC4635397

[3] Brenner E and Hall D . Computed tomography—an increasing source of radiation exposure. N Eng J Med. 2007;357(22):2277–84. 10.1056/NEJMra072149 18046031

[4] Brenner DJ and Elliston CD . Estimated radiation risks potentially associated with full‐body screening. Radiology. 2004;232(3):735–38. 15273333 10.1148/radiol.2323031095

[5] Pearce MS , Salotti JA , Little MP , et al. Radiation exposure from CT scans in childhood and subsequent risk of leukaemia and brain tumours: a retrospective cohort study. Lancet. 2012;380(9840):499–505. 22681860 10.1016/S0140-6736(12)60815-0 PMC3418594

[6] Mathews JD , Forsythe AV , Brady Z , et al. Cancer risk in 680,000 people exposed to computed tomography scans in childhood or adolescence: data linkage study of 11 million Australians. BMJ. 2013;346:2360. 10.1136/bmj.f2360 PMC3660619 23694687

[7] Joemai RMS , Geleijns J , Veldkamp WJH . Development and validation of a low dose simulator for computed tomography. Eur Radiol. 2010;20(4):958–66. 19789877 10.1007/s00330-009-1617-x PMC2835638

[8] The Phantom Laboratory. Catphan 500 and 600 phantom manual. Salem, NY: The Phantom Laboratory; 2006.

[9] Solomon JB , Christianson O , Samei E . Quantitative comparison of noise texture across CT scanners from different manufacturers. Med Phys. 2012;39(10):6048–55. 23039643 10.1118/1.4752209

[10] Saunders R Jr. and Samei E . Resolution and noise measurements of five CRT and LCD medical displays. Med Phys. 2006;33(2):308–19. 16532935 10.1118/1.2150777

[11] Armstrong JS and Collopy F . Error measures for generalizing about forecasting methods: empirical comparisons. Int J Forecasting. 1992;8(1):69–80.

[12] Singh S , Kalra MK , Hsieh J , et al. Abdominal CT: comparison of adaptive statistical iterative and filtered back projection reconstruction techniques. Radiology. 2010;257(2):373–83. 20829535 10.1148/radiol.10092212

[13] Boedeker KL , Cooper VN , McNitt‐Gray MF . Application of the noise power spectrum in modern diagnostic MDCT: part 1. Measurement of noise power spectra and noise equivalent quanta. Phys Med Biol. 2007;52(14):4027–46. 17664593 10.1088/0031-9155/52/14/002

[14] Samei E and Flynn MJ . An experimental comparison of detector performance for direct and indirect digital radiography systems. Med Phys. 2003;30(4):608–22. 12722813 10.1118/1.1561285

